# Quantifying the responses of biological indices to rare macroinvertebrate taxa exclusion: Does excluding more rare taxa cause more error?

**DOI:** 10.1002/ece3.2798

**Published:** 2017-02-08

**Authors:** Zhengda Yu, Hui Wang, Jiao Meng, Mingsheng Miao, Qiang Kong, Renqing Wang, Jian Liu

**Affiliations:** ^1^ Institute of Environmental ResearchShandong UniversityJinanChina; ^2^ College of Life ScienceShandong Normal UniversityJinanChina; ^3^ College of Geography and EnvironmentShandong Normal UniversityJinanChina; ^4^ School of Life SciencesShandong UniversityJinanChina

**Keywords:** benthic macroinvertebrate, biological indices, rare taxa, subsampling, suppositional plot simulation

## Abstract

Including or excluding rare taxa in bioassessment is a controversial topic, which essentially affects the reliability and accuracy of the result. In the present paper, we hypothesize that biological indices such as Shannon–Wiener index, Simpson's index, Margalef index, evenness, BMWP (biological monitoring working party), and ASPT (Average Score Per Taxon) respond differently to rare taxa exclusion. To test this hypothesis, a benthic macroinvertebrate data set based on recent fifteen‐year studies in China was built for suppositional plot analyses. A field research was conducted in the Nansi Lake to perform related analyses. The results of suppositional plot simulations showed that Simpson's index placed more weight on common taxa than any other studied indices, followed by Shannon–Wiener index which remained a high value with the exclusion of rare taxa. The results indicated that there was not much of effect on Simpson's index and Shannon–Wiener index when rare taxa were excluded. Rare taxa played an important role in Margalef index and BMWP than in other indices. Evenness showed an increase trend, while ASPT varied inconsistently with the exclusion of rare taxa. Results of the field study also indicated that rare taxa had few impacts on the Shannon–Wiener index. By examining the relationships between the rare taxa and biological indices in our study, it is suggested that including the rare taxa when using BMWP and excluding them in the proposed way (e.g., fixed‐count subsampling) to calculate Shannon–Wiener index and Simpson's index could raise the efficiency and reduce the biases in the bioassessment of freshwater ecosystems.

## 1 Introduction

Benthic macroinvertebrates which respond to a wide range of stressors have been increasingly studied (Menezes, Baird, & SoaresBeyond, 2010). Compared with direct physical‐chemical measurements, biological data can support assessments of long‐term ecological status (Jaehnig & Cai, 2010). Due to the human disturbances, including agriculture, industry, channelization, construction, and species introduction (Kuzmanović et al., 2016; LaBonte, Scott, McIver, & Hayes, 2001), abundant but vulnerable insects such as Ephemeroptera, Plecoptera and Trichoptera have turned into rare taxa, becoming indicators of the ecosystem health assessment (Pinel‐Alloul, Méthot, Lapierre, & Willsie, 1996).

Many researchers have paid attention to rare taxa (Jiang, Song, Xiong, & Xie, 2014) while also wondering if rare taxa could be removed during subsampling (Chen, Hughes, & Wang, 2015). Gauch (1982) suggested that rare species added noise to the statistical solution. Using common taxa to interpret patterns of disturbance or ecosystem degradation is current method of bioassessment (Marchant, 2002). However, Cao, Larsen, and Thorne (2001) argued that sample size and the rules for excluding rare species before conducting multivariate analyses need to be evaluated carefully for their unintended influences on the outcome of the analysis. Poos and Jackson (2012) summarized different viewpoints between inclusion and exclusion of rare taxa, supporting that better justifications for the removal of rare species are needed to move bioassessment forward. However, previous studies mostly focused on the responses of multivariate analyses to inclusion and exclusion of rare taxa (Cao et al., 2001; Poos & Jackson, 2012). Although biological indices are used in many methods of bioassessment, for example multi‐metric indices (Chen et al., 2014), studies on responses of biological indices to rare taxa exclusion are seldom reported. Emphases of indices are different, and some indices put more weight on common species, while some do not. We therefore hypothesize that biological indices respond differently to rare taxa exclusion.

Before testing our hypothesis, a data set with more species than even a natural “high diversity” site is needed to allow generalization and simulation. Obviously, few current data sets meet these requirements. To overcome the difficulty of simulations, a newly formed data set of benthic macroinvertebrates in China is utilized in this study. The data set based on fifteen‐year (2001–2015) data was used to examine the responses of classical diversity indices and macroinvertebrate‐based indices to rare taxa exclusion at several levels of rarity and several sizes of fixed‐count. According to classic species–area relationship (SAR, Arrhenius, 1921), the number of rare taxa would increase (or decrease) with the increased (or decreased) sample size. Sampling in field research and subsampling in the laboratory are the determinations of the number of rare taxa. We excluded rare taxa on the levels of rarity stepwise to simulate the shrinkage of sample size. We also randomly selected fixed number of individuals from total sample to simulate the fixed‐count subsampling. In order to test our hypothesis in a real natural world, a field research in Nansi Lake was also conducted. Our work offers a new perspective to other researchers by using a large‐scale sample size accompanied by a field research which is never used before to test the responses. This work may simplify procedures that are used during field sampling and subsampling and direct future efforts to develop bias‐reduction sampling methods for biological indices.

## 2 Materials and Methods

### 2.1 Published data collection

**Figure 1:**
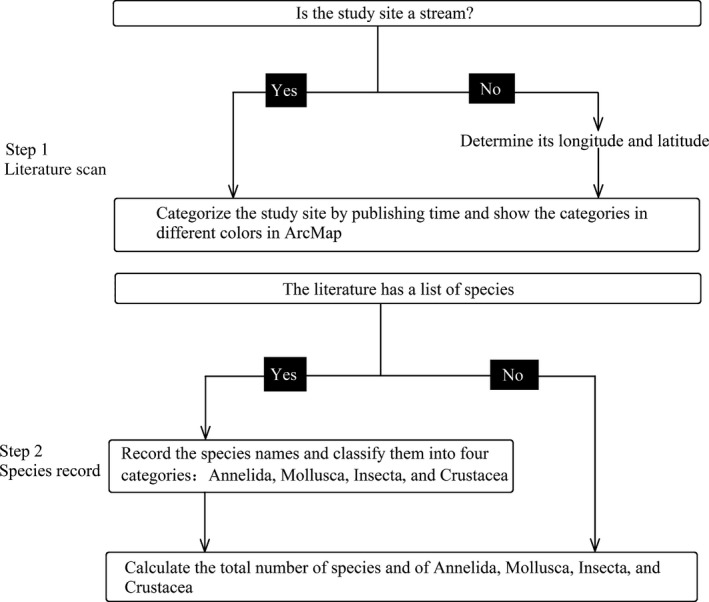
Method of published data collection

The data set originated from published scientific literature on macroinvertebrate in the last fifteen years (2001–2015) in China. First, we screened the Web of Knowledge for all papers about benthic macroinvertebrates in China published between 2001 and 2015. All papers were collected by a search using the combined terms [zoobenthos] and [China] or [macroinvertebrate] and [China]. Combining these approaches yielded a total of 1,115 papers. After the manual elimination of research on marine and estuary ecosystems, remained papers were chosen as our sample population. Second, the chosen studies were classified based on the period of publication and type of ecosystems in order to collect the detailed information for further analyses (Figure 1). A total of 374 studied sites in 219 papers were displayed chronologically on a map using ArcGIS 10.2. Among the 374 studied sites, 220 sites with detailed taxa information were selected as the origin of the data set (Appendix S1) which was considered as a “suppositional plot” in the following analyses. The suppositional plot was consisted of all taxa occurred in the data set, including three groups of taxa: Annelida, Insecta, and Mollusca. The “frequency” of taxa presented in the data set was regarded as the “abundance” of the suppositional plot. For example, *Branchiura sowerbyi* was presented in 133 times in different sites, and then the abundance of *Branchiura sowerbyi* was considered as 133. Crustacea was not listed on the inventory because it was not recorded in most studies. The data of our field research were not contained in the data set because all data set was based on the published data.

### 2.2 Current study situation

**Figure 2:**
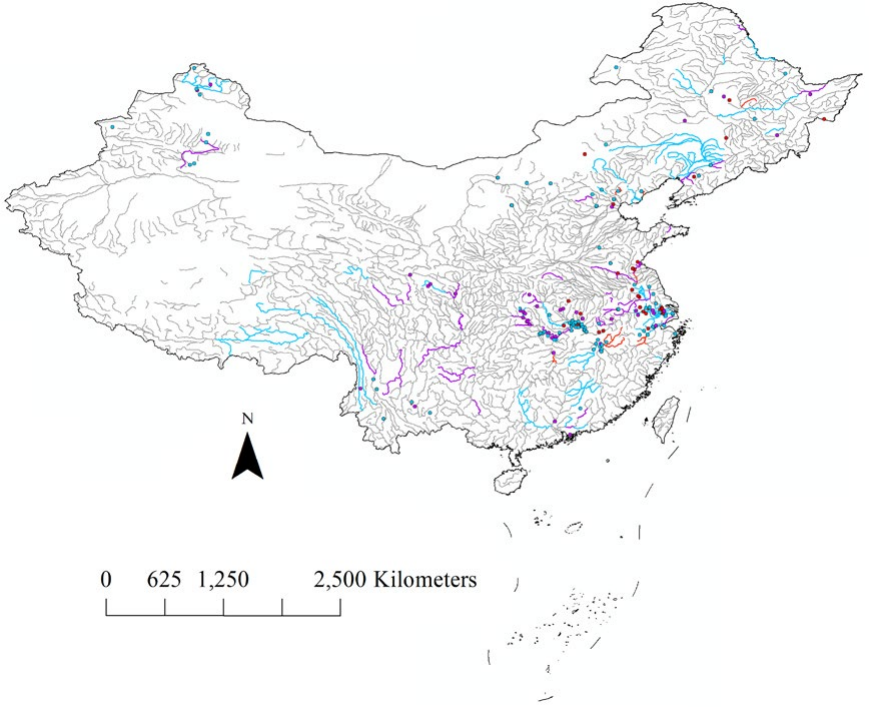
Total number of studies on macroinvertebrates in China (2001–2015). Thick lines: studied rivers and streams; solid circles: studied lakes, reservoirs, and constructed wetlands, red: 2001–2005, purple: 2006–2010, blue: 2011–2015; thin gray lines: unstudied areas

A total of 219 published papers and 374 sites were recorded over the fifteen‐year period. There were 40, 70, and 109 studies published in the first, second, and third five‐year periods, respectively. In the first five years, most studies were conducted in eastern China (Figure 2). During the second five‐year period, the number of studies mainly increased in southern China, especially studies on headwater in plateau (Figure 2). Besides, there were several studies on lakes in arid region of northwestern China (Figure 2). The increase trend kept accelerating during the recent five‐year period (2011–2015). The total number of studies in this period (*n *=* *109) nearly equaled the sum of the previous ten years (*n *=* *110).

### 2.3 Rare taxa exclusion and subsampling simulation

According to the review of bioassessment methods in collected papers, diversity indices were much more popular than other methods in the past 15 years in China. Diversity indices such as the Shannon–Wiener index, the Simpson's index, the Margalef index, and evenness (Table 1) were calculated from the total sample (true value) in this study. Biological monitoring working party (BMWP) score which has been published as a standard method by an international panel (ISO‐BMWP, 1979) is a simple, rapid but not common index in China. We wondered if it would respond differently to the exclusion of rare taxa from diversity indices. Hence, biological monitoring working party (BMWP) and Average Score Per Taxon (ASPT), which were macroinvertebrate‐based qualitative indices, were also involved in the calculation (Table 1). The BMWP score is calculated by summing the scores for each family represented in the sample. The ASPT indicates the average sensitivity of the families and can be determined by dividing the BMWP score by the number of taxa present.

**Table 1:** Six biological indices applied in the simulations

Indices	Calculation method	References
Shannon–Wiener index (*H*′)	$-\sum_{i=1}^{S}p_{i}\ln\left(p_{i}\right)$	Shannon (1948)
Simpson's index	$1-\sum_{i=1}^{S}{p_{i}}^{2}$	Simpson (1949)
Margalef index	$\frac{S-1}{\text{ln}N}$	Margalef (1958)
Evenness	$\frac{H^{\prime }}{H_{\max}^{\prime }}$	Ricotta and Avena (2003)
BMWP	Summing the scores for each family (Appendix S3)	ISO‐BMWP (1979)
ASPT	$\frac{\text{BMWP}}{S}$	

*S*, the number of species; *N*, the number of individuals in a population or community; *n*, the number of individuals in a sample from a population; *p*_*i*_ **= *n*_*i*_/*N*;* n*_*i*_, the number of individuals of species *i* in a sample from a population; $H_{\max}^{\prime }=\text{ln}S$.

**Figure 3:**
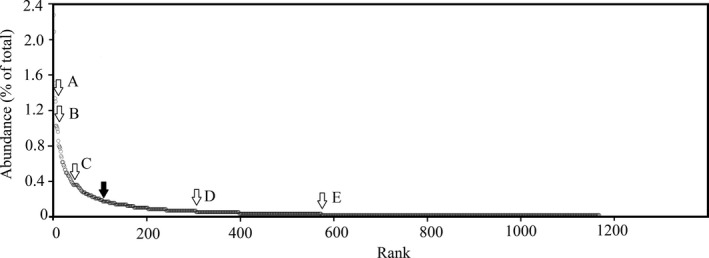
Rank–abundance curves of total sample. Abundances are expressed as percentage of the total abundance. Hollow arrows indicate the demarcation of tested criterions. (A) Occurrence frequency of <2%; (B) occurrence frequency of <1%; (C) inflection point criterion; (D) the 25% with the largest geographic distribution; and (E) 0.1–l% of the total number of individuals. Solid arrow indicates the demarcation used in this paper. Taxa at the left side of the solid arrow were classified as common, and those at the right side were classified as rare

Two types of simulations were conducted for the exclusion of rare taxa. In the first simulation, we excluded taxa stepwise on the level of rarity, calculated the indices of remained taxa, and compared the simulated value with the true value. Defining the rare taxa is the most important part in this simulation. At present, various criterions exist to define common and rare species (Table 2). We ranked all taxa with their frequency and tested all possible criterions for defining rare taxa (Figure 3). Taxa of which frequency was lower than 10 were defined as rare taxa in this study (Figure 3). The reasons why a medium value was considered as the demarcation of rare taxa in this study were as follows: (1) an excess of taxa would be excluded following A and B; (2) lacking output could be gained following D and E; and (3) C might be the most reasonable selection among the five criterions; however, based on our pre‐analysis, the output was still a little more than our expectation. Considering that moderate shifts in relative abundance do not affect the general conclusion (Magurran 2004), ten is the best demarcation of rare taxa in this study. The taxa of which frequency was more than 10 were defined as common taxa, and their details are given in Appendix S2.

**Table 2:** Criterion for defining common and rare taxa

References	Criterion	Defined taxa
Gauch (1982)	Occurrence frequency of <5%–10% of all samples	Rare
Marchant, Barmuta, and Chessman (1994)	0.1%–l% of the total number of individuals	Rare
Cao et al. (1998)	Occurrence frequency of <1%	Rare
	Occurrence frequency of <2%	Rare
	Occurrence frequency of <5%	Rare
Pärtel, Moora, and Zobel (2001)	Occurrence at more than 75% of surveyed sites	Common
Larsen, Bladt, and Rahbek (2007)	The 25% with the largest geographic distribution	Common
Siqueira et al. (2012)	Inflection point criterion	Common

**Figure 4:**
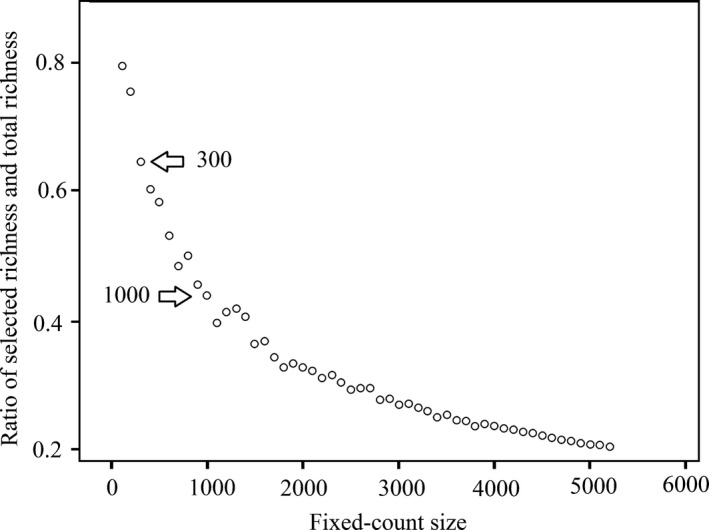
Ratio of selected richness and total richness in different fixed‐count sizes

Subsampling is used as an effective way to limit sampling error and reduce workload in a wide range of subjects, including benthic macroinvertebrate sampling (Barbour & Gerritsen, 1996; Doberstein, Karr, & Conquest, 2000; Keen et al., 2014; Petkovska & Urbanič, 2010; Petreman, Jones, & Milne, 2014; Sovell & Vondracek, 1999; Wood & Wilmshurst, 2016). But little attention is paid to subsampling in the laboratory in China. Therefore, fixed‐count subsampling was conducted in the second simulation. In this simulation, taxa were randomly selected from the total sample under different fixed‐count sizes following the selection method in Appendix S4. Because the high richness in the suppositional plot is not available for most studies at site scale and the size of fixed‐count was usually settled at 100–300 individuals (Barbour, Gerritsen, Snyder, & Stribling, 1999; Plafkin, Barbour, Porter, & Hughes, 1989), we conducted a pre‐analysis to determine the demarcation by using the inflection point criterion. Sizes from 100 to 5,200 in increments of 100 were randomly selected from total sample. The ratio of selected richness and total richness showed significant inflection at 300 and 1,000 individuals (Figure 4). Therefore, the sizes of fixed‐count simulation were determined from 300 to 1,000 in increments of 100. The random selection was conducted 30 repetitions at each increment to compensate for the bias suppositional plot.

### 2.4 Field research

A field study was conducted in Nansi Lake (116°34′E‐117°21′E, 34°27′N‐35°20′N) in the Shandong Province, China (Appendix S5). The Shandong Province is characterized by a temperate monsoon climate, with an average annual precipitation of 600 mm and a mean temperature between 13.5°C and 15°C. Nansi Lake has a total surface water area of 1,226 km^2^ and the catchment area of 31,700 km^2^. The collection of macroinvertebrates was performed using the STAR‐AQEM methodology (AQEM Consortium, 2002). The STAR‐AQEM sampling method is based on a multi‐habitat scheme designed for sampling major habitats proportionally according to their presence within a sampling boundary. A total of 12 sites located in different surrounding environments were selected as sampling sites. Macroinvertebrates were collected at all sites using a Peterson sampler (5L) annually during the wet season from 2011 to 2015. Due to the low water level, two dry sites were not available in 2015. One sample consists of 5 “replicates” taken from all microhabitat types at the sampling sites. Each sample was washed and sieved through a 40‐mesh nylon membrane. The retained materials were preserved in 75% alcohol in plastic bottles and transported to the laboratory to enumerate. All individuals were counted and identified to the lowest taxonomic level according to the relevant references (Brinkhurst, 1986; Dudgeon, 1999; Epler, 2001; Morse, Yang, & Tian, 1994; Wiggins, 1996; Zhou, Gui, & Zhou, 2003).

A total of 58 samples with taxa richness ranging from 6 to 32 at each site were collected during the field research. According to work of Cao, Williams, and Williams (1998), the indices of disturbed sites will not significantly vary when rare taxa are excluded because few rare taxa exist in these sites. However, we wondered how indices respond to the exclusion of rare taxa in undisturbed sites. Different from the suppositional plot and Cao's work (1998), we did not mix all richness and abundance of 58 samples together to define the rare taxa but excluded the rarest taxa in each site, and then the second rarest and so on. One of the drawbacks of this method is common taxa will be excluded in disturbed sites where no rare taxa appear. Hence, the results of potential disturbed sites should not be involved in further analyses. Referring to the number of common taxa (*n *=* *15) in work of Cao et al. (1998) and the average number of rare taxa per site (*n *=* *15) in Heino's work (2008), the richness in site ranging from 16 to 30 is considered as a reasonable scope for our analyses. Samples in these sites were calculated in order to explore the percentage of simulated values (simulated value/true value). In tests of effects of exclusion of rare taxa using the data of his part, the most distinctive index (Shannon–Wiener index) would be used, aiming to avoid the same results of simulation using that data of the suppositional plot. We defined true value_95%_ was an acceptable simulated value for applicable usage in normal bioassessment.

## 3 Results

### 3.1 Suppositional plot analysis

**Figure 5:**
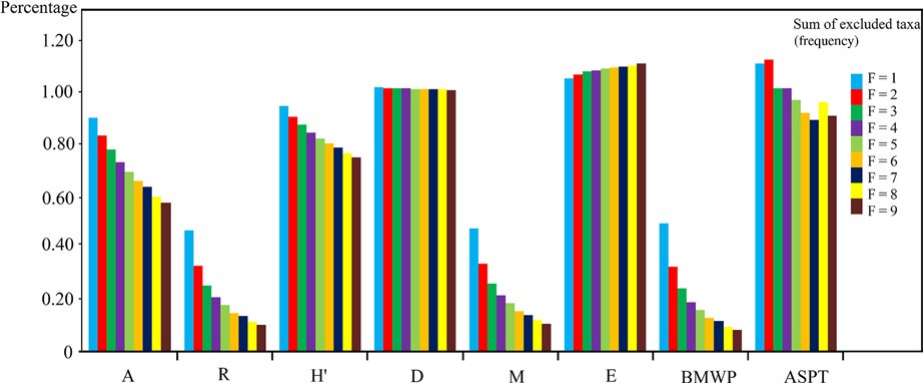
Percentage of simulated value (simulated value/true value) versus rare taxa exclusion. A, abundance; R, richness; *H* ^′^, Shannon–Wiener index; D, Simpson index; M, Margalef index; E, evenness; BMWP, biological monitoring working party; ASPT, Average Score Per Taxon

A total of 1,168 macroinvertebrate taxa with 5,217 abundance were recorded in the data set. The Shannon–Wiener index, Simpson's index, Margalef index, and BMWP showed similar trends as rare taxa were excluded. The results of the simulation indicated the variations of the BMWP (48.7%–8%) and the Margalef index (46.5%–10.5%) were most similar to that of richness (45.9%–9.8%, Figure 5). The Shannon–Wiener index steadily decreased from 5.72 to 4.54 (Table 3), and the Simpson's index changed very little. With the exclusion of rare taxa, the simulated value of evenness increased and surpassed the true value. Different from the other indices, no consistent tendency could be found in ASPT (Figure 5).

**Table 3:** Responses of biological indices to exclusion of rare taxa

Frequency of excluded taxa	1	≤2	≤3	≤4	≤5	≤6	≤7	≤8	≤9
Abundance	4,622	4,270	3,994	3,738	3,543	3,363	3,258	3,058	2,941
Richness	536	377	291	238	204	170	154	129	115
Shannon–Wiener index	5.72	5.47	5.28	5.11	4.97	4.84	4.77	4.62	4.54
Simpson's index	1.00	0.99	0.99	0.99	0.99	0.99	0.99	0.99	0.99
Margalef	63.40	44.98	34.97	28.81	24.84	20.81	18.91	15.95	14.27
BMWP	2,293	1,505	1,121	884	739	601	538	432	376
ASPT	4.89	4.96	4.47	4.47	4.26	4.06	3.94	4.24	4.00
Evenness	0.9	0.92	0.92	0.93	0.93	0.94	0.94	0.95	0.95

**Figure 6:**
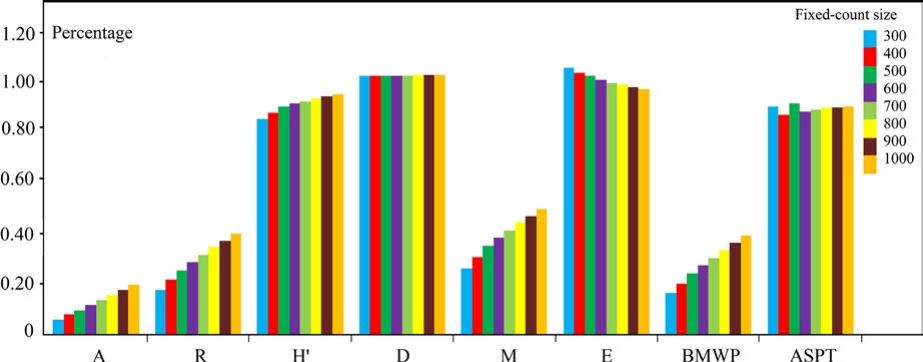
Percentage of simulated value (simulated value/true value) versus fixed‐count size. A, abundance; R, richness; *H* ^′^, Shannon–Wiener index; D, Simpson's index; M, Margalef index; E, evenness; BMWP, biological monitoring working party; ASPT, Average Score Per Taxon

As expected, the mean cumulative taxa richness increased with increasing fixed‐counts and with the other four indices: Shannon–Wiener index, Simpson's index, Margalef index, and BMWP score (Table 4). The growing rates of the BMWP (15.9%–38.2%) score and the Margalef index (20.7%–48%) were the most similar indices to that of taxa richness (17.2%–39%, Figure 6). The Shannon–Wiener index showed a slight increase from 83% to 92%, whereas the Simpson's index was almost unchanged (Figure 6). Conversely, the evenness declined with the increase of the size, with the evenness values from the 300 and 400 counts higher than the true values (103% and 101%, respectively). Different from other indices, the ASPT fluctuated irregularly with the changes in the number of counts (Figure 6).

**Table 4:** Responses of biological indices to fixed‐count size (repetition = 30)

Fixed‐count (fraction) size	300	400	500	600	700	800	900	1,000	5,217
Richness	201	246	291	326	359	395	425	456	1,168
*SD*	7.83	7.23	10.25	9.77	8.83	10.30	10.68	12.98	
Shannon–Wiener index	5.10	5.27	5.40	5.47	5.53	5.61	5.65	5.70	6.15
*SD*	0.06	0.04	0.06	0.05	0.04	0.04	0.04	0.04	
Simpson's index	0.99	0.99	0.99	0.99	0.99	0.99	0.99	0.99	1.00
*SD*	0.00	0.00	0.00	0.00	0.00	0.00	0.00	0.00	
Margalef index	35.09	40.88	46.72	50.86	54.71	59.01	62.32	65.81	136.34
*SD*	1.37	1.21	1.65	1.53	1.35	1.54	1.57	1.88	
BMWP	751.27	933.80	1,118.00	1,252.37	1,393.03	1,543.96	1,665.34	1,797.93	4,712
*SD*	49.26	51.08	66.96	63.90	63.36	57.03	58.19	58.66	
ASPT	3.94	3.80	3.99	3.84	3.88	3.92	3.92	3.94	4.48
*SD*	0.15	0.16	0.21	0.15	0.14	0.09	0.10	0.11	
Evenness	0.90	0.88	0.87	0.85	0.84	0.84	0.83	0.82	0.87
*SD*	0.01	0.01	0.01	0.01	0.01	0.01	0.01	0.01	

### 3.2 Field research

**Figure 7:**
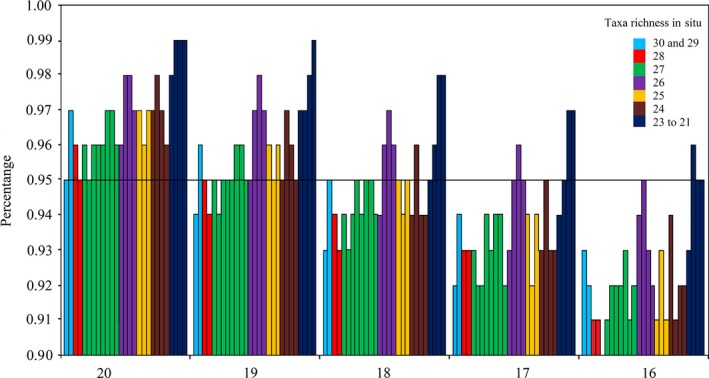
Percentage of simulated value (simulated value of Shannon–Wiener index/true value of Shannon–Wiener index) versus simulated richness

According to the result of suppositional plot simulation, we decided to calculate Shannon–Wiener index of field research data (Appendix S6). The percentages of simulated values (simulated value of Shannon–Wiener index/true value of Shannon–Wiener index) gradually decreased with the exclusion of rare taxa. In a high‐level taxa exclusion condition (10 taxa excluded), the percentages were all equal to or greater than 95% in the sites with the richness ranging from 21 to 30 (Figure 7).

## 4 Discussion

Over 200 studies on benthic macroinvertebrate in freshwater ecosystems were carried out and showed an increase in investigations of macroinvertebrates over the fifteen‐year period (Figure 2). During the progress of ecosystem restoration, researchers attempted to seek more integrated measures to assess the level of recovery. Apparently, research in eastern China, where many freshwater ecosystems have suffered from various damages, played a leading role due to the abundant water sources and anthropogenic activities (Wang, Shen, Niu, & Liu, 2009; Ye, Li, Zhang, & Zhang, 2011). Along with increasing research, the streams and lakes on plateaus in the southwestern China (Cao et al., 2016; Wang, Cai, Tang, Yang, & Li, 2012) even the headwater on the Tibet plateau (Jiang, Xie, et al. 2014; Meng, Jiang, Xiong, Wu, & Xie, 2016; Wu, Zhang, & Wang, 2015) were involved in these sorts of studies in recent five years. As found in our analysis, it is an optimistic situation that the distribution of the studies became more even.

According to our simple review, the methodology in early studies on macroinvertebrates was poorly described or even neglected by researchers and sampling methods were discrepant in China. A standard sampling method, for example Hughes and Peck (2008), listed a range of details for sampling along with other researchers (Buss et al., 2015; Li, Liu, Hughes, Cao, & Wang, 2014), is an important step in the study, and should be generalized in China. By using standard methods, the data could become more interchangeable, and the sampling error could be reduced (Chen et al., 2015). We inferred that subsampling had been used in much Chinese research because of the numerous macroinvertebrate individuals and extensive counting work, whereas the absence of detailed descriptions of the methodologies made the results hardly standardized. Moreover, fixed‐count subsampling is an efficient method, although shortcomings still exist; for example, the subsamples should be performed in a random condition (Chen et al., 2015), but are not completely randomly selected during subsampling. The increase of the calculated indices attributed to the deliberate selection of rare species usually leads to decreases in sensitiveness during subsampling. The advantage of rare taxa exclusion during subsampling in our research shields the contrived treatment for rare taxa.

Both the rare taxa exclusion simulation and fixed‐count simulation had a relative high *H* ^′^ (Tables 3 and 4) in suppositional plot. Besides, the percentage of the *H* ^′^ surpassed 90%, while counts occupied 20% of the total sample (Figure 6), indicating that there were few effects of rare taxa on *H* ^′^. Meanwhile, results of Nansi Lake further confirmed that excluding rare taxa had fewer effects on the *H* ^′^ (Figure 5). Evenness, which was calculated based on the *H* ^′^, has the same advantages. Changes in the Simpson's index could hardly be identified, which indicated that Simpson's index placed more weight on common taxa than other indices. The responses of BMWP and the Margalef index which neglected the abundance in the sample showed the most similar trend to taxa richness. In addition, the scores of the BMWP were affected by community composition. If the community was dominated by tolerant taxa, the variation amplitude of the BMWP score was higher than dominated by intolerant taxa during rare taxa exclusion because the intolerant taxa usually had high scores than tolerant taxa. The exclusion of rare taxa might cause more bias by using the BMWP score in disturbed ecosystems. In contrast, the little change in ASPT showed that the average score dilutes the bias caused by rare taxa exclusion. The determination of inclusion or exclusion of rare taxa is a debatable topic in bioassessment. Cao et al. (1998) addressed that rare taxa are critical for bioassessment because they demonstrate more ecological information, but Marchant (2002) believed conservation or protection is the major task for rare taxa which should not be involved in bioassessment. Many quantitative and half quantitative indices such as diversity indices and multi‐metrics indices are popular for bioassessment in China currently, while qualitative indices are not common. We chose BMWP as one of the simulated indices because it is a widely recognized qualitative index around the world, which avoids families of macroinvertebrates as biological indicators and avoided the need of identification of every rare macroinvertebrate taxa. At present, there is still a large amount of area unstudied area remaining in China (Figure 2), while no rapid bioassessment method exists. A widespread, common, and rapid bioassessment method to interpret patterns of ecosystem degradation which can adapt to the Chinese national condition is urgently needed. However, most of unstudied area located upstream of the aquatic ecosystem with less disturbance than well‐studied region (Figure 2). Based on our results, a combination of inclusion and exclusion rare taxa is recommended. BMWP scoring can be used during the field research before samples being preserved in containers. With the advantages of simplicity and rapidity, no taxa are needed to be excluded in this procedure. Calculation of Shannon–Wiener index and Simpson's index, which are less effected by rare taxa, only needs the details of common species. It could greatly reduce the workload in the laboratory.

However, there are also other problems in the process of bioassessment. Firstly, because the climatic conditions and disturbance levels were significantly different from site to site, the surrounding environment would undoubtedly cause various effects on the macroinvertebrate community structure. In our study, only the feature of community structure but no environmental variables was considered. Rare taxa accounted for a large portion of the total richness and abundance in the suppositional plot, which means that excluding rare taxa might blindly cause unmeasurable and confounding errors.

In conclusion, our study provides a critical test for the responses of biological indices to rare macroinvertebrate taxa exclusion. Responses of indices vary from each other when rare taxa are excluded. Our study indicates that including the rare taxa when using rapid qualitative index (BMWP) and excluding them in the proposed way (e.g., fixed‐count subsampling) to calculate diversity indices (Shannon–Wiener index and Simpson's index) could raise the efficiency, reduce the workload, and avoid biases in the bioassessment of freshwater ecosystems.

## Conflict of interest

None declared.
