# Supplementary material for: Quantifying the responses of biological indices to rare macroinvertebrate taxa exclusion: Does excluding more rare taxa cause more error?
Source: Ecol Evol. 2017 Feb 8;7(5):1583–91. doi: 10.1002/ece3.2798 (PMC5330898; doi:10.1002/ece3.2798)
Supplement: Supplementary file 2 [file ECE3-7-1583-s002.docx]

Appendix S2 List of common taxa (F=frequency)

| Annelida | F | Mollusca | F | Insecta | F |
| --- | --- | --- | --- | --- | --- |
| *Branchiura sowerbyi* | 133 | *Corbicula fluminea* | 84 | *Chironomus* *plumosus* | 77 |
| *Limnodrilus hoffmeisteri* | 122 | *Bellamya aeruginosa* | 71 | *Procladius sp.* | 58 |
| *Limnodrilus grandisetosus* | 50 | *Bellamya purificata* | 60 | *Chironomus sp.* | 56 |
| *Limnodrilus sp.* | 47 | *Radix swinhoei* | 59 | *Polypedilum sp.* | 35 |
| *Tubifex tubifex* | 43 | *Semisulcospira cancellata* | 54 | *Cryptochironomus sp.* | 33 |
| *Tubifex sinicus* | 40 | *Alocinma longicornis* | 46 | *Pelopia sp.* | 31 |
| *Aulodrilus pluriseta* | 36 | *Parafossarulus striatulus* | 44 | *Clinotanypus sp.* | 30 |
| *Limnodrilus claparedeianus* | 33 | *Unio douglasiae* | 44 | *Procladius choreus* | 30 |
| *Nais sp.* | 23 | *Limnoperna lacustris* | 39 | *Microchironomus sp.* | 29 |
| *Hirudinea sp.* | 21 | *Anodonta woodiana* | 30 | *Propsilocerus akamusi* | 29 |
| *Rhyacodrilus sinicus* | 21 | *Parafossarulus eximius* | 28 | *Ceratopogonidae sp.* | 27 |
| *Aulodrilus sp.* | 20 | *Cipangopaludina chinensis* | 27 | *Chironomidae sp.* | 27 |
| *Glossiphonia sp.* | 19 | *Sphaerium lacustr* | 27 | *Ephemera sp.* | 27 |
| *Glossiphonidae sp.* | 18 | *Bellamya quadrata* | 25 | *Glyptotendipes sp.* | 26 |
| *Tubificidae sp.* | 17 | *Radix auricularia* | 25 | *Polypedilum scalaenum* | 25 |
| *Aulodrilus pigueti* | 16 | *Radix ovata* | 22 | *Einfeldia sp.* | 23 |
| *Glossiphonia complanata* | 16 | *Cristaria Plicata* | 21 | *Hydropsyche sp.* | 21 |
| *Limnodrilus udekemianus* | 16 | *Hippeutis cantori* | 21 | *Tanypus chinensis* | 21 |
| *Tubifex sp.* | 15 | *Stenothyra glabra* | 21 | *Tanypus sp.* | 21 |
| *Lumbriculus variegatum* | 14 | *Gyraulus convexiusculus* | 20 | *Tanytarsus sp.* | 20 |
| *Nais variabilis* | 14 | *Hippeutis umbilicalis* | 20 | *Cricotopus sp.* | 19 |
| *Teneridrilus mastix* | 14 | *Cipangopaludina cathayensis* | 18 | *Ephemerella sp.* | 19 |
| *Whitmania pigra* | 14 | *Bellamya sp.* | 17 | *Baetis sp.* | 18 |
| *Dero digitata* | 13 | *Bithynia fuchsiana* | 17 | *Gomphus sp.* | 16 |
| *Monopylephorus limosus* | 13 | *Anodonta arcaeformis* | 15 | *Neureclipsis sp.* | 16 |
| *Bothrioneurum vejdovskyanum* | 12 | *Anodonta sp.* | 15 | *Dicrotendipes sp.* | 15 |
| *Nephtys oligobranchia* | 12 | *Lamprotula leai* | 15 | *Tanypus punctipennis* | 14 |
| *Aulodrilus limnobius* | 10 | *Radix plicatula* | 15 | *Tokunagayusurika akamusi* | 13 |
| *Glossiphonia lata* | 10 | *Corbicula largillierti* | 14 | *Ecdyrus sp.* | 12 |
| *Lumbriculus sp.* | 10 | *Hyriopsis cumingii* | 14 | *Tipulidae sp.* | 12 |
| *Nematoda sp.* | 10 | *Cipangopaludina sp.* | 13 | *Baetidae sp.* | 11 |
| *Nereis japonica* | 10 | *Gyraulus compressus* | 13 | *Epeorus sp.* | 11 |
|  |  | *Radix sp.* | 13 | *Stictochironomus sp.* | 11 |
|  |  | *Corbicula nitens* | 12 | *Tipula sp.* | 11 |
|  |  | *Radix lagotis* | 12 | *Cricotopus trifasciatus* | 10 |
|  |  | *Semisulcospira amurensis* | 12 | *Dytiscidae sp.* | 10 |
|  |  | *Hippeutis sp.* | 11 | *Glyptotendipes* | 10 |
|  |  | *Novaculina chinensis* | 11 | *Gomphidae sp.* | 10 |
|  |  | *Viviparus chui* | 11 | *Parachironomus sp.* | 10 |
|  |  |  |  | *Simulium sp.* | 10 |
