# Supplementary material for: Quantifying the responses of biological indices to rare macroinvertebrate taxa exclusion: Does excluding more rare taxa cause more error?
Source: Ecol Evol. 2017 Feb 8;7(5):1583–91. doi: 10.1002/ece3.2798 (PMC5330898; doi:10.1002/ece3.2798)
Supplement: Supplementary file 3 [file ECE3-7-1583-s003.docx]

Appendix S3 The biological scores allocated to groups of organisms by the Biological Monitoring Working Party (BMWP) Score

| 10 | Siphlonuridae, Heptageniidae, Leptophlebiidae, Ephemerellidae, Potamanthidae, Ephemeridae Taeniopterygidae Leuctridae, Capniidae, Perlodidae, Perlidae, Chloroperlidae, Aphelocheiridae, Phryganeidae, Molannidae, Beraeidae, Odontoceridae, Leptoceridae, Goeridae, Lepidostomatidae, Brachycentridae, Sericostomatidae |
| --- | --- |
| 8 | Astacidae,Lestidae, Agriidae, Gomphidae, Cordulegasteridae, Aeshnidae, Corduliidae, Libellulidae, Psychomyiidae (Ecnomidae), Phylopotamidae |
| 7 | Caenidae,Nemouridae, Rhyacophilidae (Glossosomatidae), Polycentropodidae, Limnephilidae |
| 6 | Neritidae, Viviparidae, Ancylidae (Acroloxidae), Hydroptilidae, Unionidae, Corophiidae, Gammaridae (Crangonyctidae), Platycnemididae, Coenagriidae |
| 5 | Mesovelidae, Hydrometridae, Gerridae, Nepidae, Naucoridae, Notonectidae, Pleidae,  Corixidae Haliplidae, Hygrobiidae, Dytiscidae (Noteridae), Gyrinidae, Hydrophilidae  (Hydraenidae), Clambidae, Scirtidae, Dryopidae, Elmidae, Hydropsychidae, Tipulidae, Simuliidae, Planariidae (Dogesiidae), Dendrocoelidae |
| 4 | Baetidae, Sialidae, Pisicolidae |
| 3 | Valvatidae, Hydrobiidae (Bithyniidae), Lymnaeidae, Physidae, Planorbidae, Sphaeriidae, Glossiphoniidae, Hiruadinidae, Erpobdellidae, Asellidae |
| 2 | Chironomidae |
| 1 | Oligochaeta |
