# Supplementary material for: Quantifying the responses of biological indices to rare macroinvertebrate taxa exclusion: Does excluding more rare taxa cause more error?
Source: Ecol Evol. 2017 Feb 8;7(5):1583–91. doi: 10.1002/ece3.2798 (PMC5330898; doi:10.1002/ece3.2798)
Supplement: Supplementary file 4 [file ECE3-7-1583-s004.docx]

Appendix S4 Detailed steps of random selection by EXCEL

1. Selection was performed by Excel and detail procedure was showed as follows: Display all 5217 sample in from A1 to A5217.

2. In put ‘=RAND()’ in B1 : randomly assign value from 0 to 1 in B1. Fill B2 to B5217 in same form.

3. In put ‘=RANK(B1,$B$1:$B$5197)’ in C1 and fill C2 to C5217 in same form: rank all species from 1 to 5217 in random order.

4. In put (1,ROWS($C$1:$C$5197)),RANDBETWEEN(1,COLUMNS($C$1:$C$5197))) in D1 and fill D2 to D5217 in same form : randomly select a number from 1 to 5217 from column C and get corresponding species in column D.
