# Supplementary figures and images for: Quantifying the responses of biological indices to rare macroinvertebrate taxa exclusion: Does excluding more rare taxa cause more error?
Source: Ecol Evol. 2017 Feb 8;7(5):1583–91. doi: 10.1002/ece3.2798 (PMC5330898; doi:10.1002/ece3.2798)

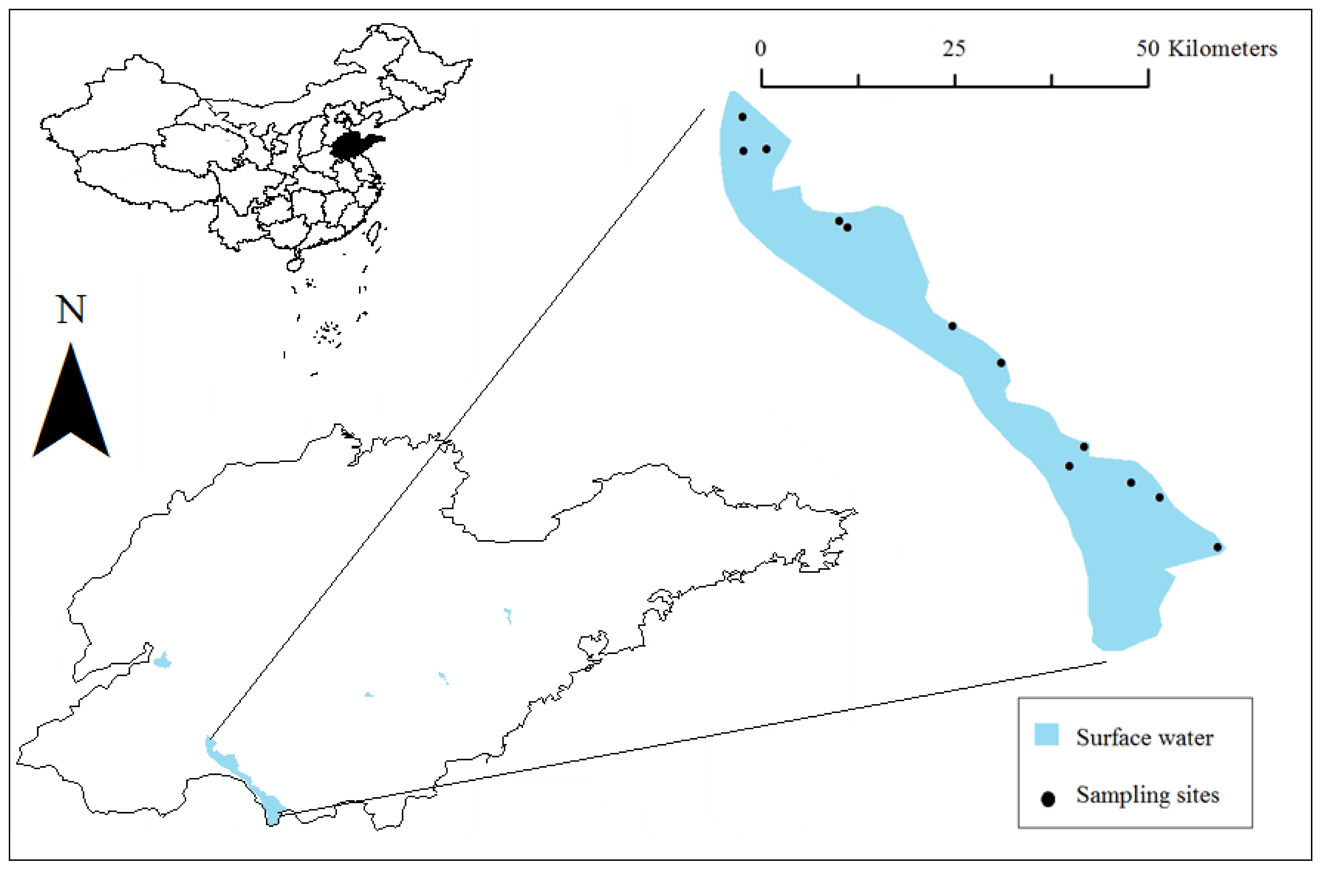

Supplement: Supplementary file 5 [file ECE3-7-1583-s005.tiff]
