# Supplementary material for: Quantifying the responses of biological indices to rare macroinvertebrate taxa exclusion: Does excluding more rare taxa cause more error?
Source: Ecol Evol. 2017 Feb 8;7(5):1583–91. doi: 10.1002/ece3.2798 (PMC5330898; doi:10.1002/ece3.2798)
Supplement: Supplementary file 6 [file ECE3-7-1583-s006.docx]

Appendix S6 Percentage of simulated value (simulated value of Shannon-Wiener index/true value of Shannon-Wiener index) versus simulated richness. Highlighted cells were data origin of figure 7.

|  | Excluding taxa following the low frequency criterion | | | | | | | | | | | | |
| --- | --- | --- | --- | --- | --- | --- | --- | --- | --- | --- | --- | --- | --- |
|  | 1 | 2 | 3 | 4 | 5 | 6 | 7 | 8 | 9 | 10 | 11 | 12 | 13 |
| 16 | 0.99 | 0.98 | 0.96 | 0.94 | 0.9 | 0.87 | 0.83 | 0.79 | 0.74 | 0.68 | 0.6 | 0.51 | 0.4 |
| 16 | 0.99 | 0.97 | 0.94 | 0.91 | 0.88 | 0.84 | 0.8 | 0.75 | 0.69 | 0.63 | 0.56 | 0.47 | 0.37 |
| 17 | 1 | 0.99 | 0.99 | 0.98 | 0.96 | 0.93 | 0.91 | 0.87 | 0.83 | 0.79 | 0.74 | 0.67 | 0.58 |
| 18 | 0.99 | 0.98 | 0.97 | 0.95 | 0.93 | 0.91 | 0.89 | 0.87 | 0.84 | 0.8 | 0.75 | 0.7 | 0.62 |
| 18 | 0.99 | 0.98 | 0.96 | 0.94 | 0.92 | 0.9 | 0.88 | 0.84 | 0.8 | 0.75 | 0.7 | 0.63 | 0.55 |
| 19 | 0.99 | 0.98 | 0.97 | 0.96 | 0.94 | 0.92 | 0.9 | 0.87 | 0.83 | 0.8 | 0.75 | 0.69 | 0.62 |
| 19 | 0.99 | 0.98 | 0.96 | 0.95 | 0.94 | 0.93 | 0.91 | 0.88 | 0.86 | 0.84 | 0.79 | 0.74 | 0.68 |
| 20 | 0.99 | 0.99 | 0.98 | 0.97 | 0.96 | 0.94 | 0.93 | 0.91 | 0.89 | 0.88 | 0.85 | 0.8 | 0.75 |
| 20 | 0.99 | 0.98 | 0.97 | 0.96 | 0.94 | 0.93 | 0.91 | 0.89 | 0.86 | 0.84 | 0.81 | 0.77 | 0.73 |
| 20 | 0.99 | 0.98 | 0.97 | 0.96 | 0.95 | 0.93 | 0.91 | 0.89 | 0.86 | 0.82 | 0.78 | 0.73 | 0.68 |
| 20 | 0.99 | 0.99 | 0.98 | 0.97 | 0.96 | 0.95 | 0.93 | 0.91 | 0.89 | 0.87 | 0.84 | 0.79 | 0.74 |
| 20 | 0.99 | 0.99 | 0.98 | 0.97 | 0.96 | 0.94 | 0.93 | 0.91 | 0.9 | 0.87 | 0.84 | 0.8 | 0.76 |
| 20 | 0.99 | 0.99 | 0.98 | 0.98 | 0.97 | 0.96 | 0.95 | 0.93 | 0.92 | 0.89 | 0.86 | 0.83 | 0.79 |
| 21 | 0.99 | 0.99 | 0.98 | 0.97 | 0.95 | 0.94 | 0.92 | 0.9 | 0.87 | 0.85 | 0.82 | 0.79 | 0.75 |
| 21 | 0.99 | 0.97 | 0.96 | 0.95 | 0.93 | 0.92 | 0.9 | 0.89 | 0.87 | 0.86 | 0.84 | 0.82 | 0.79 |
| 21 | 0.99 | 0.98 | 0.98 | 0.97 | 0.96 | 0.95 | 0.94 | 0.92 | 0.91 | 0.89 | 0.87 | 0.83 | 0.79 |
| 23 | 0.99 | 0.99 | 0.98 | 0.97 | 0.95 | 0.94 | 0.92 | 0.9 | 0.88 | 0.86 | 0.83 | 0.81 | 0.78 |
| 24 | 0.99 | 0.98 | 0.98 | 0.97 | 0.95 | 0.94 | 0.93 | 0.91 | 0.9 | 0.88 | 0.85 | 0.83 | 0.8 |
| 24 | 1 | 0.99 | 0.99 | 0.98 | 0.97 | 0.96 | 0.95 | 0.94 | 0.93 | 0.92 | 0.89 | 0.87 | 0.85 |
| 24 | 1 | 0.99 | 0.98 | 0.97 | 0.96 | 0.94 | 0.93 | 0.91 | 0.9 | 0.88 | 0.85 | 0.83 | 0.8 |
| 24 | 0.99 | 0.98 | 0.97 | 0.96 | 0.95 | 0.94 | 0.93 | 0.92 | 0.91 | 0.9 | 0.89 | 0.88 | 0.86 |
| 25 | 1 | 0.99 | 0.99 | 0.98 | 0.97 | 0.96 | 0.95 | 0.94 | 0.92 | 0.9 | 0.88 | 0.86 | 0.84 |
| 25 | 1 | 0.99 | 0.98 | 0.97 | 0.96 | 0.95 | 0.94 | 0.92 | 0.91 | 0.89 | 0.87 | 0.85 | 0.83 |
| 25 | 0.99 | 0.99 | 0.98 | 0.98 | 0.97 | 0.96 | 0.95 | 0.94 | 0.93 | 0.92 | 0.91 | 0.9 | 0.88 |
| 26 | 1 | 0.99 | 0.99 | 0.98 | 0.97 | 0.96 | 0.95 | 0.94 | 0.93 | 0.92 | 0.9 | 0.89 | 0.87 |
| 26 | 1 | 1 | 0.99 | 0.99 | 0.98 | 0.98 | 0.97 | 0.96 | 0.95 | 0.94 | 0.93 | 0.91 | 0.89 |
| 26 | 1 | 1 | 0.99 | 0.99 | 0.99 | 0.98 | 0.98 | 0.97 | 0.96 | 0.95 | 0.94 | 0.92 | 0.9 |
| 26 | 1 | 0.99 | 0.99 | 0.98 | 0.98 | 0.97 | 0.97 | 0.96 | 0.95 | 0.93 | 0.92 | 0.9 | 0.88 |
| 27 | 1 | 1 | 0.99 | 0.99 | 0.98 | 0.97 | 0.96 | 0.95 | 0.94 | 0.93 | 0.91 | 0.9 | 0.88 |
| 27 | 1 | 0.99 | 0.99 | 0.98 | 0.97 | 0.96 | 0.95 | 0.94 | 0.93 | 0.92 | 0.9 | 0.89 | 0.87 |
| 27 | 1 | 0.99 | 0.99 | 0.98 | 0.97 | 0.97 | 0.96 | 0.95 | 0.94 | 0.92 | 0.91 | 0.9 | 0.88 |
| 27 | 1 | 0.99 | 0.99 | 0.98 | 0.97 | 0.97 | 0.96 | 0.95 | 0.95 | 0.94 | 0.92 | 0.91 | 0.9 |
| 27 | 1 | 0.99 | 0.99 | 0.98 | 0.98 | 0.97 | 0.96 | 0.95 | 0.94 | 0.93 | 0.92 | 0.9 | 0.88 |
| 27 | 1 | 0.99 | 0.99 | 0.99 | 0.98 | 0.98 | 0.97 | 0.96 | 0.95 | 0.94 | 0.92 | 0.91 | 0.89 |
| 27 | 1 | 1 | 0.99 | 0.99 | 0.98 | 0.98 | 0.97 | 0.96 | 0.95 | 0.94 | 0.93 | 0.92 | 0.9 |
| 27 | 1 | 0.99 | 0.99 | 0.98 | 0.98 | 0.97 | 0.96 | 0.95 | 0.94 | 0.92 | 0.91 | 0.89 | 0.88 |
| 28 | 1 | 0.99 | 0.99 | 0.99 | 0.99 | 0.98 | 0.97 | 0.96 | 0.95 | 0.94 | 0.93 | 0.92 | 0.9 |
| 28 | 1 | 0.99 | 0.98 | 0.98 | 0.97 | 0.97 | 0.96 | 0.95 | 0.94 | 0.93 | 0.93 | 0.91 | 0.9 |
| 29 | 1 | 1 | 0.99 | 0.99 | 0.99 | 0.98 | 0.98 | 0.98 | 0.97 | 0.96 | 0.95 | 0.94 | 0.93 |
| 30 | 1 | 1 | 0.99 | 0.99 | 0.99 | 0.99 | 0.98 | 0.97 | 0.96 | 0.95 | 0.94 | 0.93 | 0.92 |
| 32 | 1 | 0.99 | 0.99 | 0.98 | 0.98 | 0.97 | 0.97 | 0.97 | 0.96 | 0.95 | 0.94 | 0.94 | 0.93 |
